# Characterization of FcγRIa (CD64) as a Ligand Molecule for Site-Specific IgG1 Capture: A Side-By-Side Comparison with Protein A

**DOI:** 10.1021/acs.langmuir.2c02022

**Published:** 2022-11-23

**Authors:** Eda Capkin, Hasan Kurt, Busra Gurel, Dilan Bicak, Sibel Akgun Bas, Duygu Emine Daglikoca, Meral Yuce

**Affiliations:** † Faculty of Engineering and Natural Sciences, Sabanci University, Tuzla 34956, Istanbul, Turkey; ‡ School of Engineering and Natural Sciences, Istanbul Medipol University, Beykoz 34810, Istanbul, Turkey; § SABITA Research Institute for Health Sciences and Technologies, Istanbul Medipol University, Beykoz 34810, Istanbul, Turkey; ∥ Nanosolar Plasmonics Ltd., Gebze 41400, Kocaeli, Turkey; ⊥ SUNUM Nanotechnology Research and Application Center, Sabanci University, Tuzla 34956, Istanbul, Turkey; # ILKO ARGEM Biotechnology R&D Center, Pendik 34906, Istanbul, Turkey

## Abstract

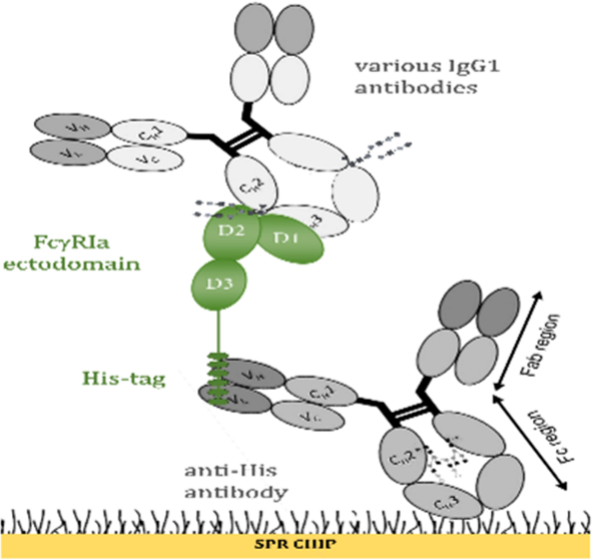

Fc γ receptors (FcγRs) are one of the structures that can initiate effector function for monoclonal antibodies. FcγRIa has the highest affinity toward IgG1-type monoclonal antibodies among all FcγRs. In this study, a comprehensive characterization was performed for FcγRIa as a potential affinity ligand for IgG1-type monoclonal antibody binding. The binding interactions were assessed with the SPR technique using different immobilization techniques such as EDC-NHS coupling, streptavidin–biotin interaction, and His-tagged FcγRIa capture. The His-tagged FcγRIa capture was the most convenient method based on assay repeatability. Next, a crude IgG1 sample and its fractions with different monomer contents obtained from protein A affinity chromatography were used to evaluate FcγRIa protein in terms of monoclonal antibody binding capacity. The samples were also compared with a protein A-immobilized chip (a frequently used affinity ligand) for IgG1 binding responses. The antibody binding capacity of the protein A-immobilized chip surface was significantly better than that of the FcγRIa-immobilized chip surface due to its 5 Ig binding domains. The antibody binding responses changed similarly with protein A depending on the monomer content of the sample. Finally, a different configuration was used to assess the binding affinity of free FcγRs (FcγRIa, FcγRIIa, and FcγRIIIa) to three different immobilized IgGs by immobilizing protein L to the chip surface. Unlike previous immobilization techniques tested where the FcγRIa was utilized as a ligand, nonimmobilized or free FcγRIa resulted in a significantly higher antibody binding response than free protein A. In this configuration, kinetics data of FcγRI revealed that the association rate (*k*_a_ 50–80 × 10^5^ M^–1^ s^–1^) increased in comparison to His capture method (1.9–2.4 × 10^5^ M^–1^ s^–1^). In addition, the dissociation rate (*k*_d_ 10^–5^ s^–1^) seemed slower over the His capture method (10^–4^ s^–1^) and provided stability on the chip surface during the dissociation phase. The *K*_D_ values for FcγRIa were found in the picomolar range (2.1–10.33 pM from steady-state affinity analysis and 37.5–46.2 pM from kinetic analysis) for IgG1-type antibodies. FcγRIa possesses comparable ligand potential as well as protein A. Even though the protein A-immobilized surface bound more antibodies than the FcγRIa-captured surface, FcγRIa presented a significant antibody binding capacity in protein L configuration. The results suggest FcγRIa protein as a potential ligand for site-oriented immobilization of IgG1-type monoclonal antibodies, and it needs further performance investigation on different surfaces and interfaces for applications such as sensing and antibody purification.

## 1 Introduction

Fc γ receptors (FcγRs) are expressed in immune cells, and they trigger various signaling cascades upon engagement with immunoglobulin (IgG) and antigen complexes, resulting in cytokine release and phagocytosis, or antibody-dependent cellular cytotoxicity (ADCC).^1,2^ Depending on their intracellular domains, FcγRs are classified as activators and inhibitors (e.g., immunotyrosine-like activation motif-ITAM or immunotyrosine-like inhibitory motif-ITIM). FcγRIa, FcγRIIα, and FcγRIIIα are activator-type receptors, whereas FcγRIIb is an inhibitory receptor that is coexpressed with other FcγRs to regulate the responses of the activator type of FcγRs. Another classification is based on their affinity to IgGs, being high- (FcγRIa) or low (FcγRIIα, FcγRIIb, FcγRIIIα)-affinity receptors.^1−3^

It has been reported that the binding between FcγRs and antibodies depends on the IgG isotypes and their glycosylation profile.^4−11^ The impact of the glycosylation profile of the monoclonal antibodies on FcγRs binding has been the core subject of many immune therapy-related reports where surface plasmon resonance (SPR) analyses were conducted to evaluate the corresponding binding characteristics.^12−14^ The interaction between IgG and FcγRs occurs through the lower hinge in the Fc region, usually with a Langmuir 1:1 binding model where one ligand molecule interacts with a single analyte molecule.^1,10,15,16^ FcγRIa is the only IgG receptor with a notably high affinity on the order of 10^–8^ and 10^–9^ M,^17^ thus vital in immunotherapy. The crystal structure of the FcγRIa extracellular domain and Fc domain of human IgG suggests a binding scheme similar to those of low-affinity FcγRII and FcγRIII receptors, with additional hydrogen bonds and salt bridges in the lower hinge region.^3,18^ The receptor D2 domain FG loop conformation also enables a unique charged KHR amino acid pattern that interacts with proximal carbohydrate units of the Fc glycans, whereas the third domain has been reported to increase specificity and affinity. Besides, it was reported that the deglycosylation of IgG1 causes an almost 40-fold loss in FcγRIa binding, highlighting the necessity of the FG loop in glycan recognition.^18^

FcγRIa comprises a transmembrane region, a cytoplasmic region, and three extracellular domains interacting with the IgGs. One unique property of FcγRIa is its high affinity for monomeric IgG, in contrast to other Fc receptors such as FcγRII and FcγRIII, which bind efficiently to the complex IgGs (dimer or aggregates).^19,20^ Despite the overwhelming amount of data published about the effector function of the FcγRIa with therapeutic monoclonal antibodies, only a limited number of studies reported the FcγRIa protein as a potential affinity ligand.^21−24^ In the study conducted by Boesch et al.,^4^ the authors developed prototypes of FcγRs-conjugated (Ia, IIa, and IIIa) affinity chromatography columns to separate IgGs of different isotypes or glycan profiles from a pooled human serum. The coupling of FcγRs was performed using EDC-NHS chemistry, which randomly constitutes a covalent bond between free carboxylic acid and primary amine groups. FcγRIIa and FcγRIIIa-coupled affinity columns accomplished the recovery of varied IgG subclasses and were further tested for their effector functions. However, the covalently coupled FcγRIa affinity column was not effective as the others due to regeneration problems. In another study by Kim et al.,^25^ FcγRIa was used to conjugate IgG-type antibodies to nanoparticles for biosensing purposes. The His-tagged FcγRIa proteins were first immobilized to the lipid-coated quantum dots using Ni-NTA conjugation chemistry. Four target-specific antibodies were later conjugated to the nanoparticles through FcγRIa–antibody interactions and evaluated further to detect cancer biomarkers, including Claudin-4, Mesothelin, Mucin-4, and Cadherin-11. FcγRIa was proposed as a universal antibody linker in this study. However, the authors did not conduct a complete analytical characterization study for the FcγRIa–antibody interaction. Despite the overwhelming amount of data published about the effector function of the FcγRIa with therapeutic monoclonal antibodies, only a few studies reported the FcγRIa protein as a potential affinity ligand with limited analytical performance information.^21−24^

Immunoglobulin G is the most widely used antibody class in many applications such as therapeutic, immunoassays, research, and diagnostic purposes. Among the IgG subtypes (IgG1, IgG2, IgG3, IgG4), IgG1 subtype is stated as the majority of the approved therapeutic monoclonal antibodies.^26^ The widespread use of IgG1 has made it necessary and important to develop methods for their production, isolation, and selection from complex samples.^27^ The detection of monoclonal antibodies is performed by either Fc binding proteins (protein A, protein G, protein A/G) or Fab binding protein L.^17,28^ Protein A could bind all IgG subtypes with a high affinity except for IgG3 subtype.^29^ However, some studies revealed that protein A, protein G, and protein A/G ligands could build nonspecific interactions with the Fab region of the antibodies.^30^ In addition to these ligands, researchers have developed alternative peptide ligands to capture IgGs on versatile surfaces.^27,31,32^ Conventional IgG detection is an enzyme-linked immunoassay (ELISA); however, it requires sequential steps and labeled secondary biomolecule for the detection.^33^ Various techniques (fluorescence, optic, electrochemical) are available for IgG detection and enhanced their sensitivity by applying surface modification and nanoparticle conjugation (gold, magnetic, quantum dots, etc.).^27,31,33−38^ An optic-based approach, surface plasmon resonance, offers many advantages such as real-time monitoring, low sample consumption, and reduced assay time.^38^ Analytical characterization of the FcγRIa as an alternative ligand molecule for site-directed IgG1 capture was performed in the current study. A systematic approach was adopted to evaluate the potential of FcγRIa as an alternative affinity ligand for IgG1-type monoclonal antibody binding. SPR technique was used to monitor and compare the binding interactions obtained from different immobilization techniques. Then, cell supernatants of a biosimilar product obtained from different purification steps were used to compare FcγRIa and protein A-immobilized surfaces for IgG1 binding. Finally, we revealed the in-solution binding affinity of free FcγRIa to IgGs. The initial results promise a bright future for FcγRIa in analytical chemistry, especially in site-oriented IgG1 capture on surfaces and interfaces for biosensing applications.

## 2 Materials and Methods

### 2.1 IgG1 Binding Capacity Analysis with FcγRIa and Protein A Used as Ligands: Reference Monoclonal Antibodies were Used as Analytes

The IgG1 binding capacity analysis of immobilized FcγRIa and protein A for three monoclonal antibodies—adalimumab (ADA), avastin (AVT), and herceptin (HER)—was carried out on a CM5-type dextran chip (Cat no: 29-1496-03, Cytiva) by applying a standard EDC/sulpho-NHS primary amine coupling procedure^39^ using a Biacore T200 SPR system (Cytiva). Later, two alternative conjugation methods were implemented.

First, His capture method was performed for FcγRIa binding analysis. An amine coupling kit was used to apply the anti-His IgG1 antibody immobilization procedure based on the manufacturer’s guide (His Capture kit, Cytiva). First, the chip surface was activated by a 1:1 mixture of EDC-NHS reagents. Then, anti-His antibody (1 mg mL^–1^) was diluted to 50 μg mL^–1^ in 10 mM sodium acetate pH 4.5 immobilization buffer and injected into the chip surface. Finally, the chip surface was blocked with 1 M ethanolamine-HCl (Cytiva) for the residual activated carboxyl groups on the dextran matrix. As a second method, the chip surface was activated by a 1:1 mixture of EDC-NHS reagents for protein A (Sigma-Aldrich, *Staphylococcus aureus*, ≥95% purity) immobilization. Then, protein A was diluted to 25 μg mL^–1^ in 10 mM pH 5.0 acetate buffer and coupled through their primary amine groups to one flow cell with a 10 μL min^–1^ flow rate at 22 °C. The residual activated carboxyl groups were blocked with 1 M ethanolamine-HCl (Cytiva) on the dextran matrix with a 30 μL min^–1^ flow rate at 22 °C. The final immobilization level for the active flow cells reached approximately 200 response units (RUs). FcγRIa (R&D Systems, NS0-derived human Fc γ RI, >95% purity) was captured on the active flow cells for 60 s with a 10 μL min^–1^ flow rate at 22 °C. Three different concentrations (10, 30, 90 nM) of monoclonal antibody samples were injected on both flow cells (active and blank) with 60 s association and 600 s dissociation with a 30 μL min^–1^ flow rate at 22 °C. The surface was regenerated with 10 mM glycine (pH 1.5) for 60 s. The SPR data were presented as the mean value obtained from at least three sample measurements. The kinetic parameters—*k*_a_, *k*_d_, and equilibrium dissociation constants (*K*_D_)—were calculated by Biacore Evaluation Software (version 3.0) using either the 1:1 Langmuir binding model (for FcγRIa) or the heterogeneous binding model (for protein A). *K*_D_ values from affinity analysis were performed with steady state by Biacore Evaluation Software. The SPR data were presented as the mean value, calculated from at least three measurements per sample.

### 2.2 IgG1 Binding Capacity Analysis with FcγRIa and Protein A Used as Ligands: Biosimilar Harvest Samples were Used as Analytes

An anti-VEGF biosimilar harvest product from the ILKO ARGEM Biotechnology R&D Center was purified with protein A affinity chromatography (GE) using an AKTA FPLC instrument. Elution and clean-in-place (CIP) fractions were also collected for analysis. The sample solution was exchanged to HBS-EP five times with a 10 kDa protein filter unit (Amicon Ultra-0.5, EMD-Millipore). Finally, the concentration of all samples was adjusted to 15 nM with 1× HBS-EP buffer.

The purity level of monoclonal antibody fractions was quantified with a size exclusion high-performance liquid chromatography (SEC) system (Waters e2695) on a TSK-GEL G3000SWxL (7.8 mm × 300 mm, Tosoh Biosciences) column. Reference sample (Avastin, AVT), a biosimilar harvest supernatant, and monoclonal antibody fractions (Elution, CIP) diluted in distilled water were loaded. Before use, all SEC-high-performance liquid chromatography (SEC-HPLC) system buffers were filtered with a poly(ether sulfone) membrane filter (0.2 μm) and degassed. The samples were monitored by ultraviolet (UV) absorbance at 280 nm. The monomeric monoclonal antibody level was obtained by determining the peak area of each species as a percentage of the total peak area.^12,40,41^

Protein A, anti-His antibody, and FcγRIa were immobilized on the CM5 chip using the amine coupling reaction on the second, third, and fourth flow cells for two different CM5 chips. FcγRIa (14 and 30 nM) was captured on the third flow cell for 60 s with a flow rate of 10 μL min^–1^ at 22 °C. Monoclonal antibody samples were injected at 15 nM for 60 s with a flow rate of 10 μL min^–1^.

The results were obtained with double referencing, where the presented response was subtracted from the zero-concentration sample (buffer) and blank surface (either naïve CM5 surface or ethanolamine-coated surface). The mean value and standard deviation were calculated from at least three measurements per sample.

### 2.3 IgG1 Binding Capacity Analysis with Protein L-Captured Antibodies as Ligands: FcγRIa, FcγRIIa, and FcγRIIIa were Used as Analytes

The binding analysis of recombinant FcγRIa, FcγRIIa, and FcγRIIIa (R&D systems) for three different monoclonal antibodies was performed with a Biacore T200 SPR system (Cytiva). Protein L (Pierce) was immobilized on two flow channels of the CM5 chip by applying a standard amine coupling reaction (Cytiva). First, the chip surface was activated by a 1:1 mixture of EDC-NHS reagents with a 30 μL min^–1^ flow rate at 22 °C. Then, protein L was diluted to 25 μg mL^–1^ in 10 mM pH 4.0 acetate buffer and coupled through their primary amine groups to two flow cells. The residual activated carboxyl groups were blocked with 1 M ethanolamine-HCl (Cytiva) on the dextran matrix. The final immobilization level for the flow cells reached approximately 300 response units (RUs). FcγRs and three monoclonal antibodies, adalimumab (AbbVie, Humira Pen, 1126059), avastin (Roche, B8703H35), herceptin (Roche, Herceptin, N7377B51U1), were prepared with 1× HBS-EP running buffer. Single-cycle kinetic analyses were conducted at a 30 μL mL^–1^ flow rate at 22 °C.^42^ Adalimumab, avastin, and herceptin at 6 nM concentrations were captured on the active flow cells for 60 s with a 10 μL mL^–1^ flow rate at 22 °C. Three different concentrations (1.66, 5, 15 nM) of FcγRIa, FcγRIIa, and FcγRIIIa samples were injected through both flow cells (active and blank) with 60 s association and 600 s dissociation with a flow rate of 30 μL mL^–1^ at 22 °C. The surface was regenerated with 10 mM glycine buffer at pH 1.5 for 60 s. Results were obtained with double referencing, subtracting the active surface response from the zero-analyte concentration sample (buffer) and blank surface (either naïve CM5 surface or ethanolamine-coated surface). The SPR data were presented as the mean value and standard deviation, calculated from at least three measurements per sample. One-way analysis of variance, ANOVA, was used to reveal the statistically significant data (*p* < 0.05 was considered significant and *p* < 0.005 was considered highly significant).

### 2.4 IgG1 Binding Capacity Analysis with Protein L-Captured Antibodies as Ligands: FcγRIa and Protein A were Used as Analytes

The binding analyses of the FcγRIa and protein A (Sigma-Aldrich) in solution were carried out on a protein L-immobilized dextran-coated CM5 chip (Cytiva). The immobilization procedure was applied as previously described in Section 2.3. FcγRIa, protein A, and selected monoclonal antibodies (adalimumab, avastin, and herceptin) were prepared with 1× HBS-EP running buffer. Single-cycle kinetic analyses were conducted at a flow rate of 30 μL min^–1^ at 22 °C. Adalimumab, avastin, and herceptin were captured on the active flow cells for 60 s with a flow rate of 10 μL min^–1^ at 22 °C. Five different concentrations (0.37, 1.11, 3.33, 10, 30 nM) of FcγRIa and protein A samples were injected on both flow cells (active and blank) with 60 s association and 600 s dissociation with a flow rate of 30 μL min^–1^ at 22 °C. The surface was regenerated with 10 mM glycine (pH 1.5) for 60 s. Results were obtained by subtracting the blank sample and reference surface signal from the active surface. The SPR data were presented as the mean value, calculated from at least three measurements per sample. One-way analysis of variance, ANOVA, revealed the statistically significant differences between the sample pairs (*p* < 0.05 was considered significant and *p* < 0.005 was considered highly significant). The equilibrium dissociation constants (*K*_D_) were calculated by Biacore Evaluation Software using a 1:1 Langmuir binding model.

## 3 Results and Discussion

### 3.1 IgG1 Binding Capacity Analysis with FcγRIa and Protein A Used as Ligands: Reference Monoclonal Antibodies were Used as Analytes

FcγRIa comprises a transmembrane region, a cytoplasmic region, and three extracellular domains interacting with the IgGs (Figure S1A). Protein A, on the other hand, consists of five immunoglobulin binding domains and binds to the CH_2_–CH_3_ region in the Fc site of the antibodies at neutral pH conditions. Antibody binding performances of these two molecules were first assessed with direct coupling of FcγRIa ectodomain and protein A on different CM5-type dextran chip channels by EDC/sulpho-NHS reaction; however, any response was obtained with the direct coupling of FcγRIa ectodomain. Therefore, two other alternative methods, biotinylated FcγRIa on the SA chip and the His capture method, were applied to evaluate FcγRIa’s monoclonal antibody binding capacity. The problem associated with FcγRIa being a ligand was the regeneration of the immobilized FcγRIa, which caused distortions in the IgG binding and lowered reproducibility of the streptavidin–biotin assays. The most stable and reproducible results were obtained with the His-tag capture method, but the data for the streptavidin–biotin capture method were also presented to share the experience.

The biotinylated FcγRIa ectodomain was evaluated on a streptavidin-coated chip surface, aiming for a site-directed immobilization of FcγRIa ectodomain to the streptavidin surface for subsequent studies (Figure S1B). SPR assays were conducted on a low consumption mode with AVT antibody at 90 nM. The optimum conditions were screened for the most stable baseline and the highest sample response by assessing many different buffer solutions. However, the binding analysis results were not reproducible. IgGs were not efficiently recovered from the FcγRIa-immobilized surface, leading to the IgGs’ accumulation on the surface and an increase in the baseline response for subsequent cycles. Therefore, only AVT was tested in the binding analysis with 100 mM phosphoric acid as the regeneration buffer for 20 cycles. The sample response decreased from 360 to 60 RU between the first and the last cycles. Also, the baseline increased gradually till the last cycle (Figure S1C,D). Similar results were also reported by Boesch et al. (2018), who conjugated the FcγRs to a chromatography resin to recover different IgG subtypes from the human serum. Elution of the IgGs was accomplished with glycine buffer, but FcγRIIa and FcγRIIIa maintained their IgG binding activity, while FcγRIa could not be used after the buffer treatment.^4^ In our study, glycine buffer also disrupted the FcγRIa structure after the first injection, and the protein could not bind the antibodies for the following cycle (data not shown).

In addition, some molecular modeling studies indicated that the glycan structure stabilizes the interaction between the FcγRIa and the IgG, and thus it is hard to disrupt the interaction without harming the ligand.^43,44^ Our findings with regeneration scouting were similar to the studies, which reported that the regeneration of FcγRIa–IgG from the chip surface was complex due to high affinity.^44^ Despite several attempts, the amine coupling method did not perform successfully for FcγRIa; it resulted in a few RU of IgG binding with considerable variations among technical repeats (Figure S2). A similar result for EDC/NHS coupling of FcγRIa was also reported in the literature.^44^ Thus, a His-Tag capture method was adopted in the study. The method was applied using a CM5 chip coated initially with an anti-His antibody for His-tagged IgG capture rather than the well-known Ni^2+^-nitriloacetic acid (NTA) chips. This approach circumvents ligand heterogeneity due to coupling, surface regeneration, or renewal.^20^ However, the NTA chips could be more efficient than the current method since they do not require the initial anti-His antibody immobilization and the His-tagged FcγRIa capture step for each sample, reducing the overall ligand cost.^45^ Eventually, further experiments were conducted with the His-tag capture method.

**Figure 1:**
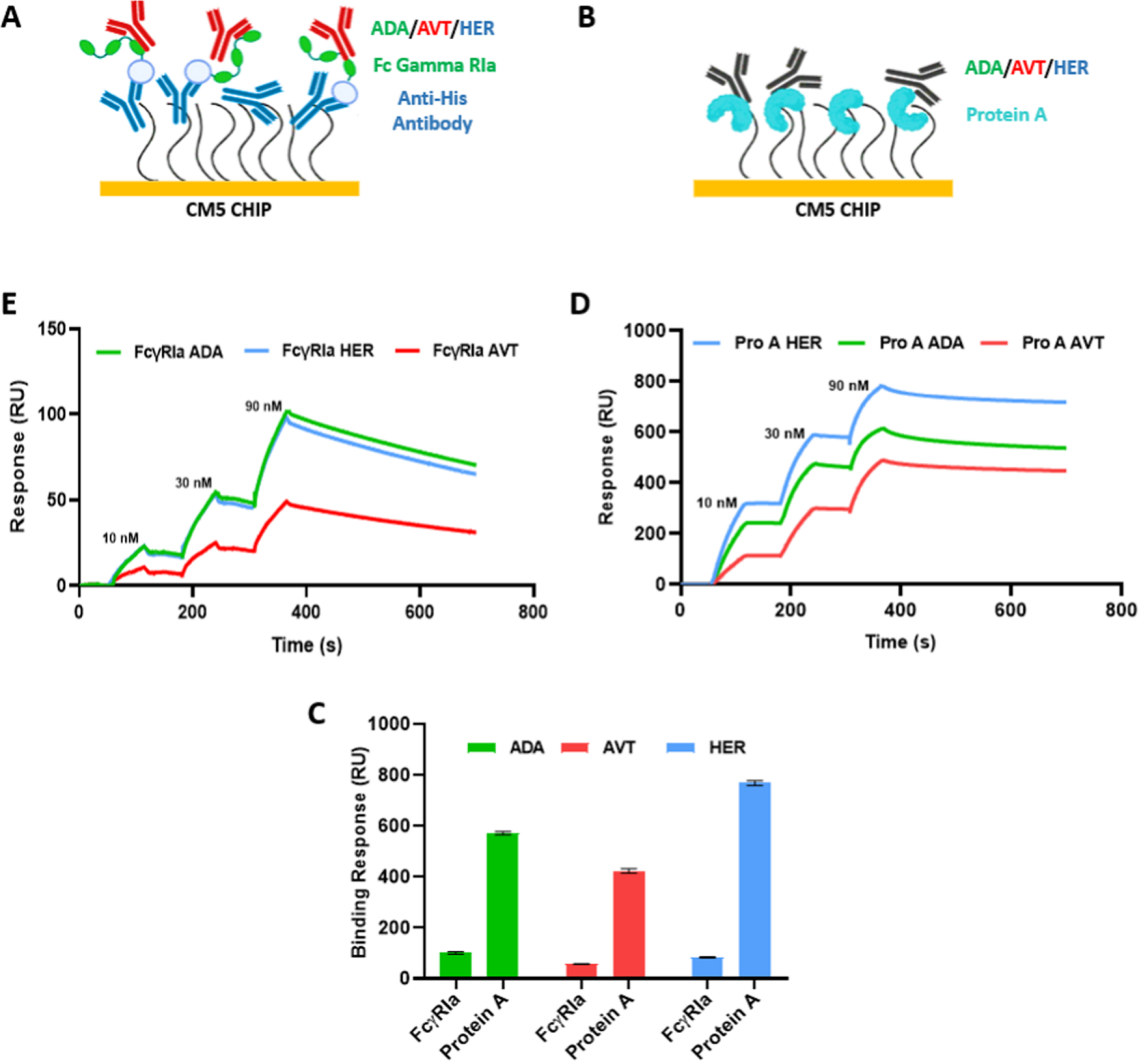
Comparison of IgG1 binding capacity with anti-His capture and direct immobilization methods for FcγRIa and protein A, respectively. (A) Schematic illustration of the anti-His and protein A binding assay on SPR CM5-type dextran chip. FcγRIa was captured on an anti-His antibody-immobilized surface. (B) Protein A was coupled by EDC/NHS conjugation chemistry. ADA, AVT, and HER were injected at three concentrations (10, 30, and 90 nM) and analyzed with a single-cycle kinetics model. The illustration was created with BioRender. (C) IgG1 binding response for FcγRIa and protein A. The data were presented as the mean value obtained from at least three measurements. (D) Representative SPR sensorgrams of protein A binding to ADA, AVT, and HER. (E) Representative SPR sensorgrams of FcγRIa binding to ADA, AVT, and HER.

In the chip configuration presented in Figure 1A,B, anti-His antibodies and protein A were directly coupled to the CM5 chip surface with EDC/NHS coupling method. FcγRIa was later captured through its His-tag at each experiment. IgG1-type monoclonal antibodies (ADA, AVT, and HER) were compared in terms of the binding response levels, and immobilized protein A and captured FcγRIa levels were kept constant at 200 RU. As presented in Figure 1C, the monoclonal antibody binding response of FcγRIa was dramatically lower than that of protein A, in sharp contrast to the in-solution binding analysis results (Sections 3.3 and 3.4), where those proteins were employed as analytes rather than ligands. However, the results were not surprising because protein A has five IgG binding domains that give rise to an interaction beyond 1:1 when used as a ligand. As reported previously, the binding stoichiometry between monoclonal antibodies and protein A was calculated at 2.4–3.1 (ratio) in a solution analysis.^46^ Also, it should be noted that the immobilization of protein A was performed through EDC-NHS reaction, whereas FcγRIa ectodomain was captured through an anti-His antibody, introducing an additional distance between the actual sensor surface and the analyte, thus lowering the signal response.

Real-time interactions of IgGs and FcγRIa displayed a fast decline at the dissociation phase for each monoclonal antibody on the anti-His antibody-immobilized surface. It is known that kinetics and affinity values could vary significantly depending on the SPR assay configuration. His capture method presented a nonstable sensorgram profile during the dissociation phase. Alternative to the His capture method, protein A, E/K coil peptides, and biotin capture studies were reported for the FcγRIa–IgG interaction analysis.^10^ ADA and HER always showed higher response levels in two data sets than AVT (Figure 1D,E). The kinetic parameters were analyzed with a 1:1 Langmuir interaction model for FcγRIa and a heterogeneous ligand model chosen for protein A (Table 1). The steady-state *K*_D_ values were in the range of 77.1–106.6 nM for FcγRIa binding analysis (Table 2). These findings were similar to the IgG–FcγRIa interaction results that were reported previously in the literature.^2,10,47,48^ Protein A sensorgrams were not globally analyzed with a 1:1 interaction due to the presence of five potential target-binding domains. The steady-state *K*_D_ values for protein A were in the range of 10.67–35.28 nM. The quantity of the antibody utilized in these experiments is usually significantly high; thus, the affinity of human IgG1 for natural FcγRIa may have been undervalued.^20^ Thus, the FcγRIa and IgG interaction is worth investigating further with complementary techniques like ELISA and bilayer interferometry. On the other hand, the structure, stability, and product yield of FcγRIa may be improved through genetic engineering techniques for analytical purposes, such as antibody purification.^15,49−51^

**Table 1:** Kinetics and Affinity Parameters Related to FcγRIa or Protein A Interactions with ADA, AVT, and HER^a^

		FcγRIa			protein A					
	sample	*k*_a_ × 10^5^ (M^–1^ s^–1^)	*k*_d_ × 10^–4^ (s^–1^)	*K*_D_ (nM)	*k*_a_1__ × 10^5^ (M^–1^ s^–1^)	*k*_a_2__ × 10^5^ (M^–1^ s^–1^)	*k*_d_1__ × 10^–4^ (s^–1^)	*k*_d_2__ × 10^–4^ (s^–1^)	*K*_D_1__(nM)	*K*_D_2__ (nM)
KINETICS	ADA	2.4 ± 0.15	9.5 ± 0.3	3.9 ± 0.1	8.1 ± 6.0	4.4 ± 5.9	4 ± 6.16	6.7 ± 5.4	13.9 ± 2	22.2 ± 2
	AVT	1.9 ± 0.19	10.4 ± 0.7	5.5 ± 0.4	12.8 ± 12	11.9 ± 13	18 ± 25.6	15.9 ± 24	0.8 ± 0.7	0.5 ± 0.8
	HER	2.4 ± 0.13	10.6 ± 0.5	4.3 ± 0.1	4.9 ± 5.9	14.4 ± 5.7	2.4 ± 1.17	0.4 ± 1.05	1.0 ± 0.5	0.16 ± 0.4

a For FcγRIa, the kinetic parameters were calculated by Biacore Evaluation Software using a 1:1 Langmuir binding model, and the heterogeneous model was utilized for protein A.

**Table 2:** Affinity Parameters Related to FcγRIa or Protein A Interactions with ADA, AVT, and HER^a^

		FcγRIa		protein A	
	sample	*R*_max_	*K*_D_ (nM)	*R*_max_	*K*_D_ (nM)
AFFINITY	ADA	169.72	78.0 ± 5.18	936.73	10.67 ± 0.52
	AVT	90.93	106.6 ± 13.81	749.40	35.28 ± 2.47
	HER	156.52	77.1 ± 6.94	1038.52	15.3 ± 0.17

a The steady-state model was utilized for the affinity values.

### 3.2 IgG1 Binding Capacity Analysis with FcγRIa and Protein A Used as Ligands: Biosimilar Harvest Samples were Used as Analytes

**Figure 2:**
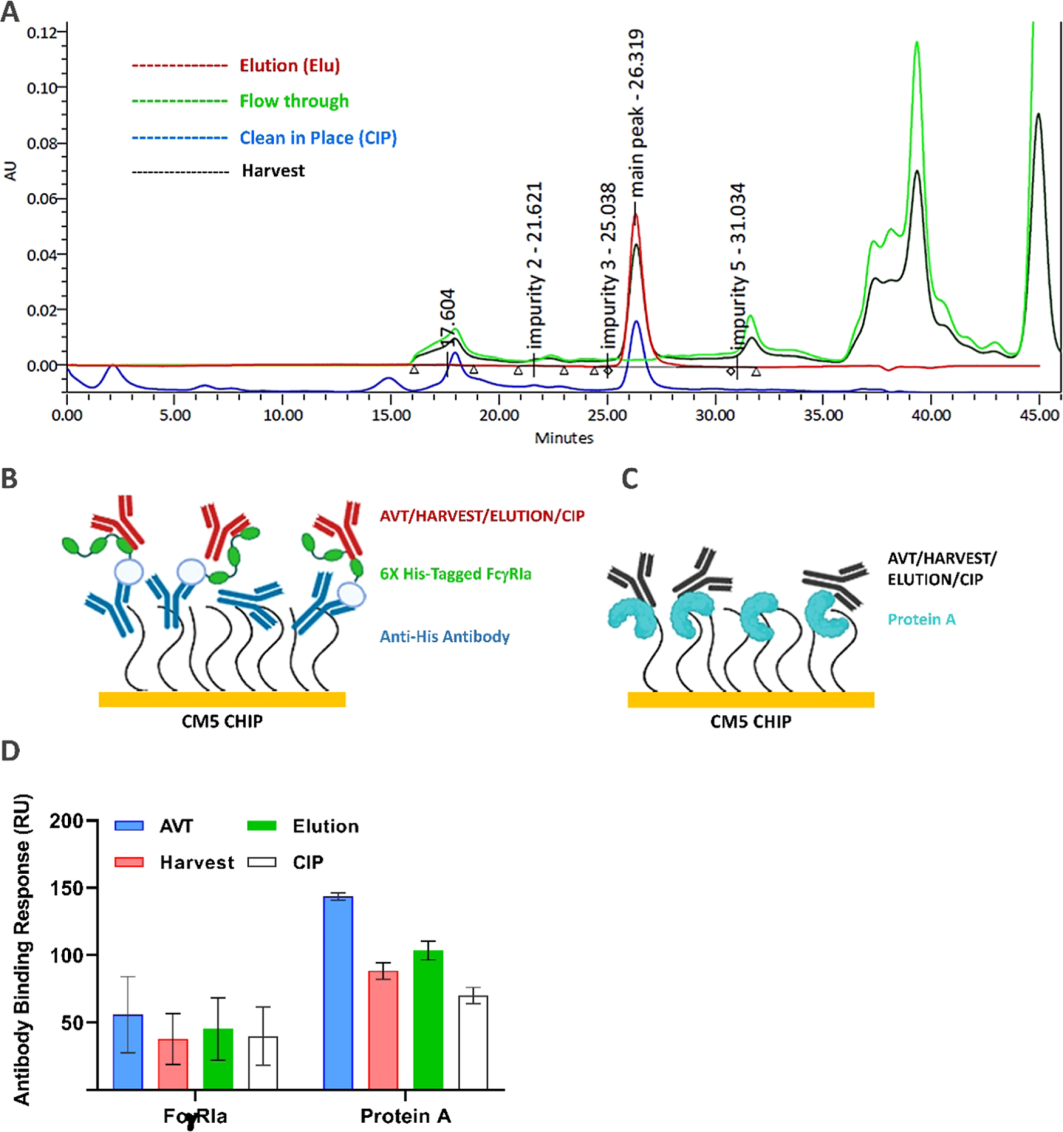
Comparative analysis of FcγRIa and protein A in terms of IgG1 and antigen-binding performance from biosimilar harvest. (A) Chromatogram profile of samples (AVT, harvest, elution, and CIP) was obtained from SEC-HPLC analysis. (B) Schematic illustration of the binding analysis with the anti-His antibody surface. FcγRIa was captured on the anti-His antibody-immobilized surface. (C) Schematic illustration of the binding analysis with protein A surface. Protein A was coupled by EDC/NHS conjugation chemistry. AVT, harvest, elution, and CIP were injected at a 15 nM concentration, and target antigen was injected at 15 nM for 60 s association and 600 s dissociation. The illustration has been created with BioRender. (D) Antibody binding responses were evaluated for AVT, harvest, elution, and CIP fractions with FcγRIa and protein A ligands. Data were presented as the mean value obtained from at least three measurements.

The IgG binding performance of FcγRIa protein was also evaluated with a biosimilar’s crude samples. For this purpose, a biosimilar candidate harvest was utilized and purified with protein A affinity chromatography to collect monoclonal antibodies with various monomer purities (elution and clean-in-place (CIP) fractions). SEC analysis was conducted to reveal the monomer content of the samples. AVT was utilized as a control reference sample with a high purity level (99%). The monomer levels were 48.50, 98.45, and 39.98% for harvest, elution, and CIP fractions, respectively (Figure 2A).

All samples were buffer-exchanged to the HBS-EP system solution and adjusted to a 15 nM concentration with the same buffer for SPR assays. The chip configuration for SPR assays was illustrated in Figure 2B,C. Protein A was directly coupled to the CM5 chip surface via EDC/NHS chemistry, and FcγRIa was captured on an anti-His antibody-immobilized surface. As stated in the Materials and Methods section, immobilized protein A and captured FcγRIa levels were kept constant at 200 and 300 RU, respectively. For the reliability of the assay, it was repeated on two different CM5 chips. Since we aimed to compare monoclonal antibody binding capacity, we checked the monoclonal antibody binding response with 200 and 300 RU surfaces. For the FcγRIa-captured surface, monoclonal antibody purity levels did not significantly alter the binding to FcγRIa. The highest binding response levels were 81.8 RU with the AVT sample (99% purity). Even in the CIP fraction with a 40% monomer IgG content, the antibody binding response was 59.9 RU. Dorion-Thibaudeau et al. (2017) performed a similar SPR analysis with FcγRIa ectodomains to examine the monoclonal antibody titer and its binding activities from the cell culture. The authors stated that the FcγRIa ectodomain maintained a stable ligand performance during SPR monitoring of monoclonal antibody samples from the harvest.^52^ As presented in Figure 2D, protein A responses were higher than that of FcγRIa. The binding to protein A surface was in correlation with the purity level of the samples. AVT sample presented a 1.77-fold higher monoclonal antibody binding response than FcγRIa. CIP fraction presented the lowest monoclonal antibody response with a value of 64.5 RU.

Next, we evaluated the FcγRs binding with IgGs in a different immobilization format, an in-solution assay, using protein L-immobilized and antibody-captured SPR surface for FcγR binding.

### 3.3 IgG1 Binding Capacity Analysis with Protein L-Captured Antibodies as Ligands: FcγRIa, FcγRIIa, and FcγRIIIa were Used as Analytes

**Figure 3:**
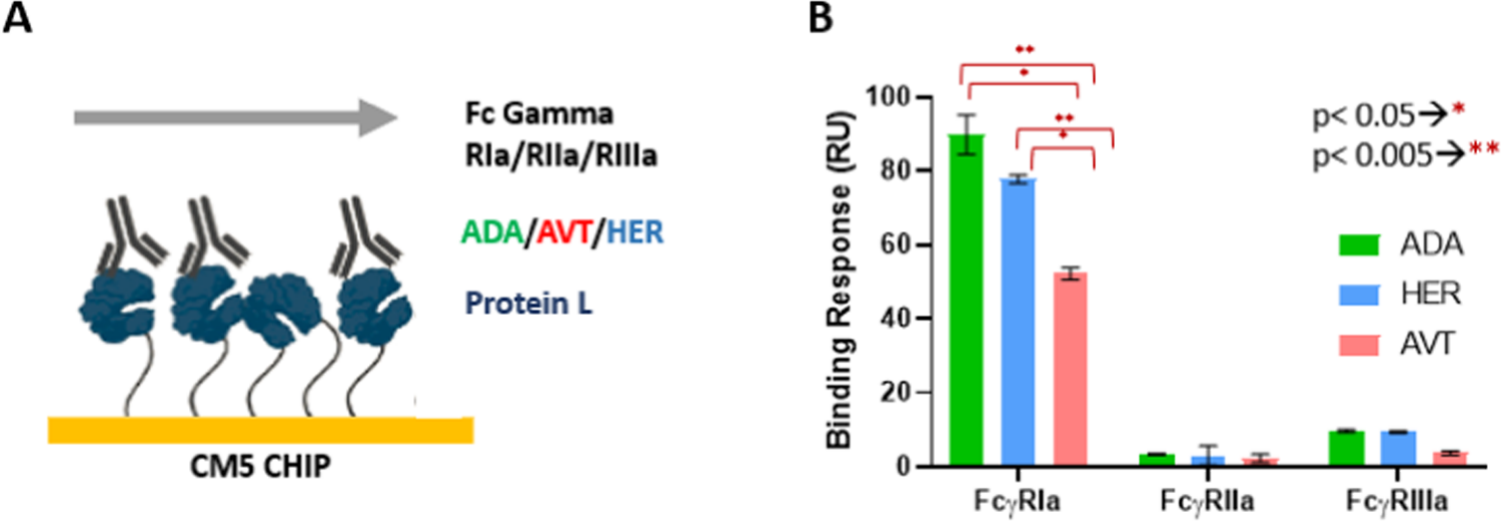
Comparison of FcγRIa, FcγRIIa, and FcγRIIIa for IgG1 binding capacity with in-solution orientation. (A) Schematic illustration of the in-solution binding assay on SPR. The ligands ADA, AVT, or HER (6 nM) were captured on a protein L-immobilized surface, and the samples (FcγRIa, FcγRIIa, or FcγRIIIa) were injected at three different concentrations (1.66, 5, 15 nM). The Illustration was created with BioRender. (B) In-solution IgG1 binding response for free FcγRIa, FcγRIIa, and FcγRIIIa. Data were presented as the mean value obtained from at least three measurements.

Various assay formats were reported in the literature to assess the affinity of monoclonal antibodies to FcγRs with SPR.^2,6,13,44,53^ SPR assays are frequently performed with amine coupling of either FcγRs or monoclonal antibodies on the chip surface, or the His-tag capture method is used to examine interactions between FcγRs and monoclonal antibodies.^10^ Here, an alternative approach was used to reveal the in-solution IgG1 binding characteristics of FcγRIa, FcγRIIa, and FcγRIIIa on the protein L-immobilized chip. Protein L binds to the kappa light chain in the Fab region of monoclonal antibodies. It is an effective ligand for an oriented capture of molecules on surfaces or particles.^54,55^ With this assay configuration, model IgG1-type monoclonal antibodies (ADA, AVT, HER) were captured on the protein L-immobilized surface through their Fab regions, and the Fc regions of the antibodies that bind to FcγRs were left exposed to the solution for target binding (Figure 3A).

The FcγRs (Ia, IIa, and IIIa) were injected onto the antibody-captured surfaces to monitor the IgG1 binding behavior of free FcγRs proteins. In Figure 3B, IgG1 binding characteristics of free FcγR proteins (used as analytes) were compared for three monoclonal antibodies (used as ligands). The highest binding response level was found with FcγRIa. The binding response levels of ADA, AVT, and HER to FcγRIa were 89 ± 5, 52 ± 2, and 77 ± 1 RU, respectively. The lowest binding response level was obtained with FcγRIIa, which was 3 ± 0.2, 2 ± 1, and 3 ± 2 RU for ADA, AVT, and HER, respectively. FcγRIIIa binding response analysis for ADA, AVT, and HER was 10 ± 0.5, 4 ± 0.5, and 9 ± 0.2 RU, respectively. The binding levels differed depending on the captured monoclonal antibodies on the protein L surface. HER mediates a mechanism of action through its Fc region resulting in ADCC activities on the target cells; ADA possesses both CDC (complement-dependent cytotoxicity) and ADCC activities.^12,56^ AVT is not capable of inducing either CDC or ADCC activity. In addition to that, the distinct glycan profile of the monoclonal antibodies probably affected the interactions with FcγRs.^18^ This is a critical quality attribute of IgGs that rely on a CDC-based mechanism. The major glycan profile of HER contains Man5, G0F,-GN, G0, G0F, G1F, and G2F.^12,57^ Predominant glycan forms of ADA are high galactose glycans, which are G0F, G1F, and G2F. Other glycan forms include afucosylated (≤ 1.7%), high mannose (<10%), and sialylated (≤0.3%).^41,58,59^ AVT contains G0F, G1F, and G2F N-glycan types. Minor glycan forms include afucosylated (≤1.7%), high mannose (≤1.3%), and sialylated (<0.2%).^40^ Several studies reported N-glycans’ effect on the FcγR–IgG interactions.^2,9,57,59−61^ Lack of core fucose (afucosylation) in the IgG structure was indicated as a main inducer for the ADCC activity, and it led to enhanced binding affinity to FcγRIIIa.^57,60^ Most therapeutic monoclonal antibodies include less than 15% afucosylation. The efficacy of ADCC or a CDC-based mechanism could be altered with engineered afucosylation levels.^60^

### 3.4 IgG1 Binding Capacity Analysis with Protein L-Captured Antibodies as Ligands: FcγRIa and Protein A were Used as Analytes for Comparison

**Figure 4:**
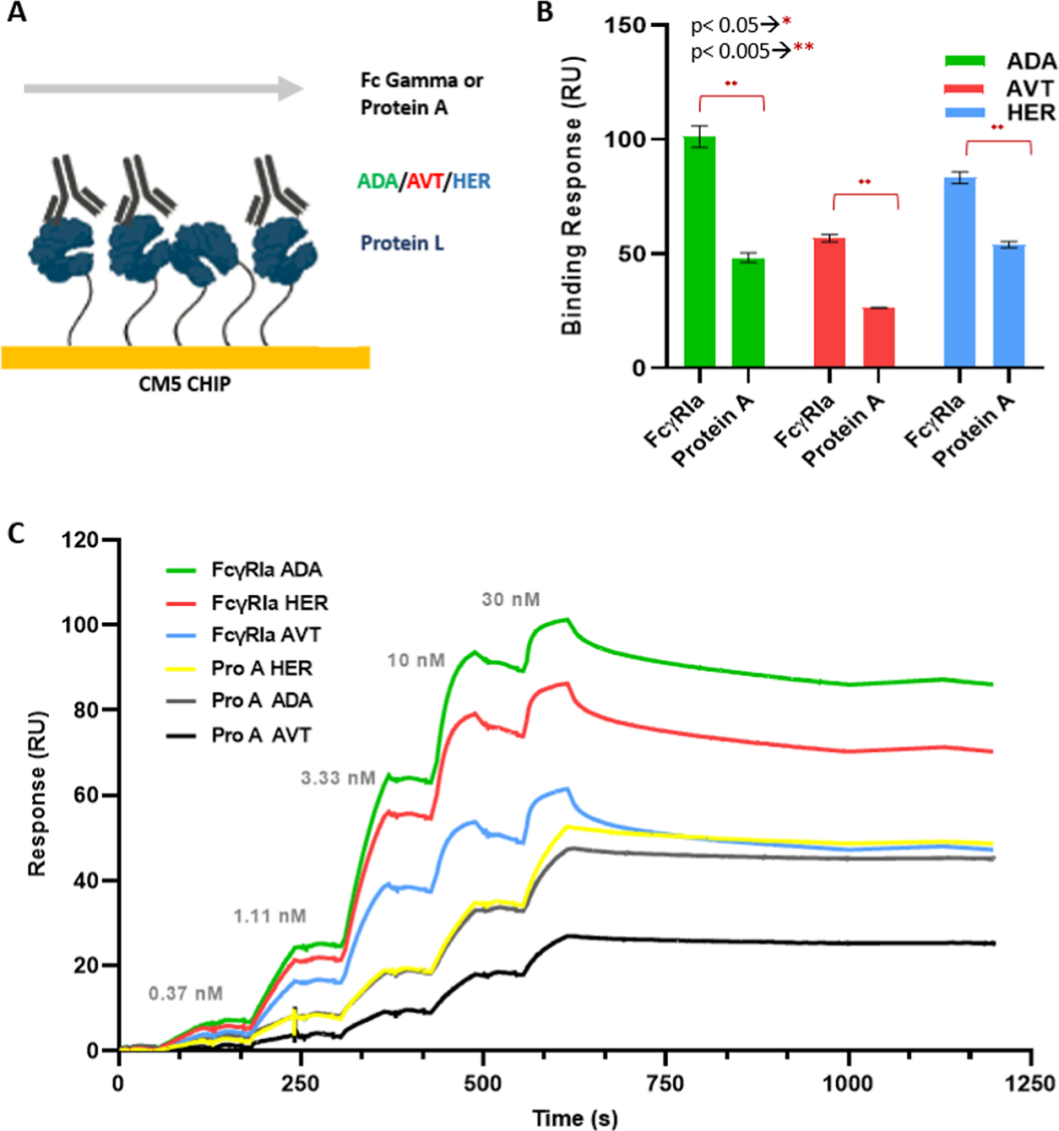
Comparison of FcγRIa and protein A for IgG1 binding with an in-solution orientation where these molecules were used as analytes instead of ligands. (A) Schematic illustration of the in-solution binding assay on SPR ADA, AVT, or HER (6 nM) was captured on protein L-immobilized surface, and the samples (FcγRIa or protein A) were injected with five concentrations (0.37, 1.11, 3.33, 10, 30 nM) using single-cycle kinetics mode. The Illustration was created with BioRender. (B) Results of in-solution IgG1 binding response for FcγRIa and protein A. Data were presented as the mean value obtained from at least three measurements. (C) Representative SPR sensorgrams of FcγRIa or protein A binding to ADA-, AVT-, or HER-captured surfaces.

Upon successful IgG1 binding performance of FcγRIa over the other Fc receptors tested, we further compared the IgG1 binding potential of free FcγRIa protein with protein A. Based on the in-solution binding kinetics results in this study (Figure 3B), further investigation of FcγRIa as an alternative ligand molecule seemed viable. First, different IgG1-type monoclonal antibodies (ADA, AVT, HER) of the same concentration (6 nM) were captured on a protein L-immobilized chip surface (Figure 4A). Then, the FcγRIa and protein A samples prepared at five different concentrations were injected onto the antibody-captured surfaces and evaluated for the final binding response at equilibrium and the binding kinetics. The antibody binding capacity of free FcγRIa and free protein A was compared for ADA, AVT, and HER binding, as presented in Figure 4B. The equilibrium binding responses of ADA, AVT, and HER were 101 ± 5, 57 ± 2, and 83 ± 3 RU for FcγRIa and 48 ± 2, 26 ± 0.2, and 54 ± 1 RU for protein A, respectively. In agreement with the previous data set, the IgG1 binding capacity of free FcγRIa was statistically more significant than that of free protein A.

On the other hand, the IgG binding capacity of FcγRIa and protein A varied for all tested antibodies, indicating a glycosylation-dependent binding variation, as previously reported by research groups.^61^ Increased concentrations of FcγRIa displayed a fast association profile in the sensorgram over the monoclonal antibody-captured surface (Figure 4C). However, the response declined over the dissociation phase. The sensorgram of protein A did not reach a saturation profile at the same concentration range for the association step, but it maintained a more stable interaction during the dissociation phase.

The kinetics and affinity parameters presented in Tables 3 and 4 were obtained using Langmuir 1:1 binding interaction model and steady-state model. In the kinetic analysis, the *k*_a_ value was found to be remarkably higher for FcγRIa (51.7–83.5 × 10^5^ M^–1^ s^–1^) than for the protein A (13 × 10^5^ M^–1^ s^–1^). However, the *k*_d_ value for protein A was almost half of that for FcγRIa. Once we take the five IgG binding sites of protein A into consideration, a lower *k*_d_ for the protein A–IgG interaction is reasonable since any IgG leaving the binding site on protein A could easily find another binding site nearby. This naturally led to a more stable interaction between the monoclonal antibody and protein A during the dissociation phase. The *K*_D_ values obtained from kinetic parameters were between 37.5 and 46.2 pM for FcγRIa and 45.1 and 103.8 pM for protein A. On the other hand, steady-state affinity values were similar for both ligands within the range of 2.1–10.3 pM.

**Table 3:** Kinetics and Affinity Parameters Related to FcγRIa or Protein A Interactions with ADA, AVT, and HER^a^

		FcγRIa			protein A		
	sample	*k*_a_ × 10^5^ (M^–1^s^–1^)	*k*_d_ × 10^–5^ (s^–1^)	*K*_D_ (pM)	*k*_a_ × 10^5^ (M^–1^ s^–1^)	*k*_d_ × 10^–5^ (s^–1^)	*K*_D_ (pM)
KINETICS	ADA	72.4 ± 10.79	27.7 ± 0.49	38.9 ± 5.68	13.2 ± 0.45	13.7 ± 0.19	103.8 ± 3.19
	AVT	51.7 ± 6.07	24.2 ± 7.60	46.2 ± 13.45	12.4 ± 0.34	6.5 ± 0.26	52.7 ± 2.51
	HER	83.5 ± 10.89	30.7 ± 5.37	37.5 ± 9.26	13.1 ± 0.65	5.0 ± 3.55	45.1 ± 20.12

a The kinetic parameters were calculated by Biacore Evaluation Software using a 1:1 Langmuir binding model.

**Table 4:** Affinity Parameters Related to FcγRIa or Protein A Interactions with ADA, AVT, and HER^a^

		FcγRIa		protein A	
	sample	*R*_max_	*K*_D_ (pM)	*R*_max_	*K*_D_ (pM)
AFFINITY	ADA	129.1	2.33 ± 0.04	59.15	9.39 ± 0.52
	AVT	73.62	2.11 ± 0.16	35.12	10.32 ± 0.33
	HER	109.12	10.03 ± 0.5	69.65	2.11 ± 0.15

a The steady-state model was utilized for the affinity values.

The FcγRIa–IgG characterization studies reported *K*_D_ values ranging from 0.1 to 100 nM with diverse immobilization strategies in which FcγRIa was usually immobilized to the surface as a ligand.^10,13,14^ Our SPR studies indicate that the *K*_D_ values vary significantly depending on the FcγRIa protein orientation and are susceptible to conjugation chemistry. The steric hindrance could partially explain this result where the orientation of the molecules on the surface may have changed the binding interactions, especially for the soluble FcγRIa ectodomain, which could easily find the Fc regions aligned on the chip. In addition, the immobilization or capture of FcγRIa onto a surface as a ligand may have disturbed its conformational structure, resulting in a decrease in IgG binding capacity. Crystallization studies for FcγRIa suggested that D3 domain within the extracellular domain provides stability and flexible orientation upon binding.^62^ In His capture assay, D3 domain contains a histidine tag and this can be limited to the FcγRIa structure for the IgG binding. In protein L configuration, FcγRIa ligand freely interacted with IgG1 and that may be the reason for high-affinity values in comparison to the previous assay format. Here, we identified the in-solution binding affinity of free FcγRIa to IgGs in the low pM range. The oriented configuration of IgGs on the protein L surface provided an equal comparison of FcγRIa and protein A for the IgG binding, where the FcγRIa presented a better performance than protein A when they were used as analytes rather than ligands.

## 4 Conclusions

Protein A is a bacterial membrane protein commonly utilized to purify monoclonal antibodies. It consists of five immunoglobulin binding domains and binds to the CH_2_–CH_3_ region in the Fc site of the antibodies at neutral pH conditions. Recovery of IgGs with protein A is obtained at acidic buffer conditions (pH: 3.0–3.5). However, there are significant issues with the protein A ligand, such as acidic elution conditions, protein A leakage, nonspecific association with impurities, and cost.^63−67^ These drawbacks of the protein A ligand have led researchers to explore new ligands, including FcγRIa, to capture and purify the monoclonal antibodies.^64,68,69^ FcγRIa has a high affinity against the Fc region of the IgGs. Due to its 1:1 binding stoichiometry, it provides a site-specific capture of IgGs without a steric hindrance. It could be a useful ligand for target antigen detection for the IgGs.

In the current study, a systematic approach was adopted to evaluate the analytical potential of FcγRIa as an alternative affinity ligand for IgG1-type monoclonal antibody binding. We implemented different surface immobilization techniques with FcγRIa being either ligand or analyte and tested three different IgG1-type commercial biosimilar monoclonal antibodies. The results showed that FcγRIa has the potential to be a capturing agent for monomeric IgG molecules, but its binding performance is significantly lower than that of protein A under the tested experimental conditions. Later, the target antibodies were captured on protein L-coated SPR chips through their Fab regions, and the corresponding FcγRIa and protein A were injected as the analytes to confirm the integrity and activity of the Fc regions. The results were the opposite: the antibody-captured chip performed significantly better regarding FcγRIa binding.

In addition, a biosimilar candidate’s crude harvest, elution, and CIP samples were tested for that assay, along with a highly pure (99%) reference AVT sample. An SEC analysis was conducted to reveal the monomer content of the biosimilar samples. As expected, the protein A surface bound significantly more antibodies than the FcγRIa-captured surface. Overall results suggest FcγRIa protein as a potential ligand for site-oriented immobilization of IgG1-type monoclonal antibodies on surfaces and interfaces, especially for antigen-sensing applications, which will be investigated further by our group in the future.

## Supporting Information Available

Characterization of biotinylated FcγRIa and AVT interaction on an SA chip (Figure S1); antibody binding responses with EDC/sulpho-NHS conjugation chemistry for FcγRIa (Figure S2), and parameters related to FcγRIa interactions with AVT in different FcγRIa ligand concentrations (Table S1) (PDF)

## Notes

All authors have read and agreed to the published version of the manuscript.
