# Supplementary material for: Characterization of FcγRIa (CD64) as a Ligand Molecule for Site-Specific IgG1 Capture: A Side-By-Side Comparison with Protein A
Source: Langmuir. 2022 Nov 23;38(48):14623–34. doi: 10.1021/acs.langmuir.2c02022 (PMC9730901; doi:10.1021/acs.langmuir.2c02022)
Supplement: Supplementary file 1 — la2c02022_si_001.pdf [file la2c02022_si_001.pdf]

Supporting Information

Characterization of FcγRIa (CD64) as a Ligand Molecule for site-specific IgG1 Capture: A Side-by-side Comparison with Protein A

Eda Capkin[1], Hasan Kurt[2,3,4], Busra Gurel[5], Dilan Bicak[6], Sibel Akgun Bas[6], Duygu Emine Daglikoca[6], Meral Yuce[5*]

[1] Sabanci University, Faculty of Engineering and Natural Sciences, 34956, Istanbul, Turkey

[2] Istanbul Medipol University, School of Engineering and Natural Sciences, 34810, Istanbul, Turkey

[3] Istanbul Medipol University, SABITA Research Institute for Health Sciences and Technologies, 34810, Istanbul, Turkey

[4] Nanosolar Plasmonics Ltd., Gebze, 41400, Kocaeli, Turkey

[5] Sabanci University, SUNUM Nanotechnology Research, and Application Center, 34956, Istanbul, Turkey

[6] ILKO ARGEM Biotechnology R&D Center, 34906, Pendik, Istanbul, Turkey

***Corresponding author:** *meralyuce@sabanciuniv.edu*

Table of Contents

Figure S1 Characterization of biotinylated FcγRIa and AVT interaction on an SA chip……………………S-3

Figure S2 Antibody binding responses were evaluated with EDC/Sulpho-NHS conjugation chemistry for FcγRIa ………..……S-4

Table S1. Parameters related to FcγRIa interactions with AVT in different FcγRIa ligand concentrations. The kinetic parameters were calculated by Biacore Evaluation Software using a 1:1 Langmuir binding model……..……S-5

An alternative method was performed with biotinylated FcγRIa (Acro Biosystems) on the streptavidin-coated chip (SA) (Cytiva) chip. For the non-covalent immobilization of the FcγRIa ligand, the procedure was applied based on the manufacturer's guide (Cytiva). Biotinylated FcγRIa was prepared in HBS-EP 1X buffer solution at 100 nM concentration. The active flow cell was conditioned with 1M NaCl in 50 mM NaOH for 60 s. Then, FcγRIa immobilized on the active flow channel at a 200 RU level. The wash step was performed with 50% isopropanol in 1 M NaCl and 50 mM NaOH. Binding analysis was performed with AVT at 30 nM concentration into both flow cells (active and blank) with 60 s association and 600 s dissociation with 30 μL·min^{-1} flow rate at 22 °C. The surface was regenerated with 100 mM phosphoric acid pH 3.0 solution for 30 s. Results were obtained with double referencing, subtracted from zero concentration samples blank surface. The SPR data is presented as the mean value, calculated from at least three measurements per sample.

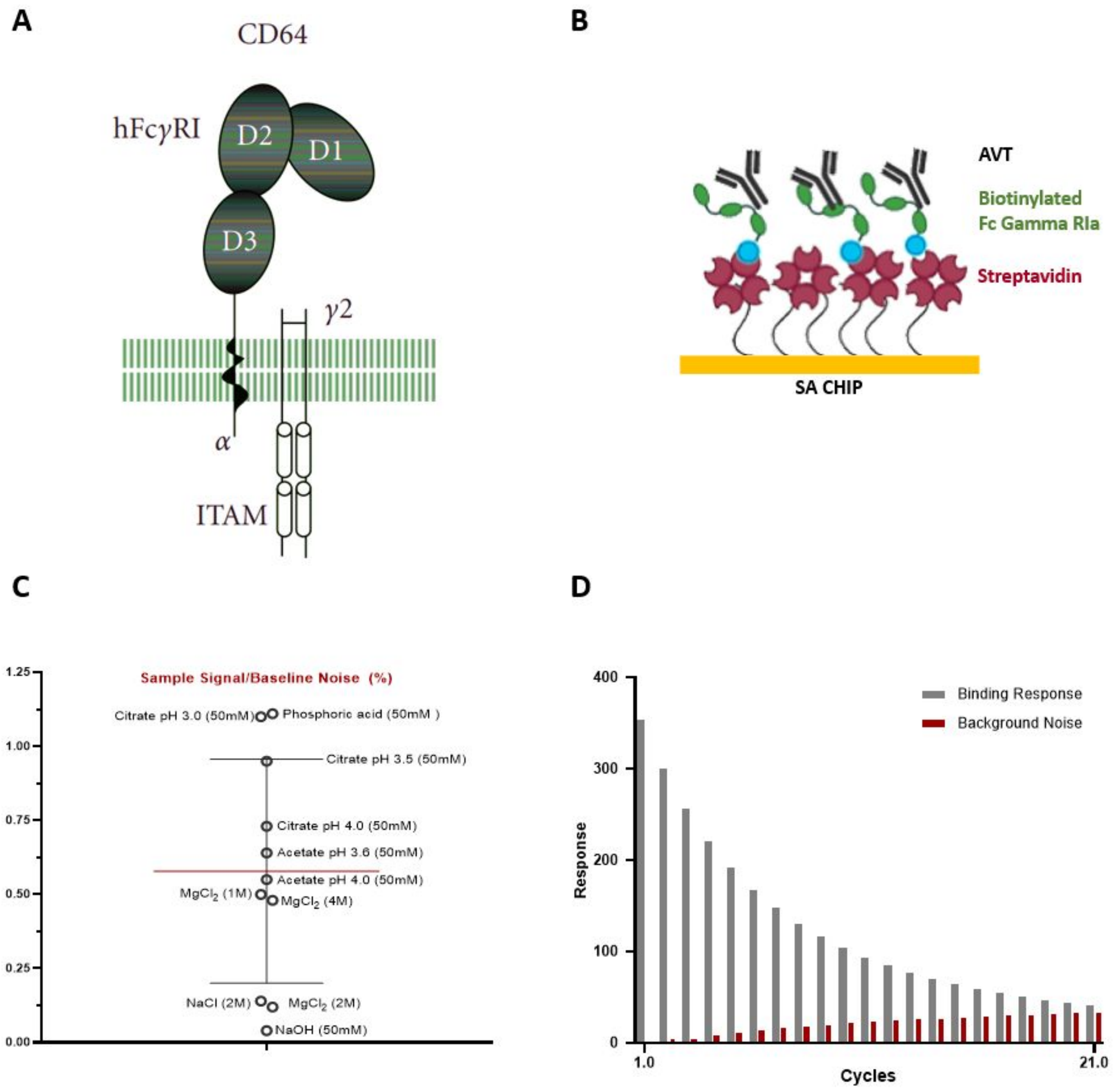

Figure S1. Characterization of biotinylated FcγRIa and AVT interaction on an SA chip A) FcγRIa structure [42], B) Schematic illustration of the biotinylated FcγRIa binding assay on an SA chip. The illustration was created with the BioRender trial version. C) Screening of various regeneration conditions for biotinylated FcγRIa. Sample (AVT) and Baseline responses were normalized based on the control run response. 50 mM pH 3.0 citrate buffer and 50 mM phosphoric acid solution seemed to have a higher sample response and less accumulated AVT on the biotinylated FcγRIa surface. D) Repeatability study performed with AVT at 30 nM concentration for 20 cycles with 100 mM phosphoric acid regeneration condition.

According to the results, the best regeneration conditions were chosen as 50 mM citrate at pH 3.0 and 50 mM phosphoric acid solution, and the binding analysis was performed with these regeneration solutions.

Amine coupling FcγRI

FcγRIa immobilized on the CM5 chip using the amine coupling reaction on the fourth flow cell. The chip surface is activated by a 1:1 mixture of EDC-NHS reagents. FcγRIa was diluted to 25 $\mu g \cdot mL^{-1}$ in 10 mM pH 5.0 acetate buffer and coupled through their primary amine groups to flow cell 4. The residual activated carboxyl groups were s blocked with 1M ethanolamine-HCl (Cytiva) on the dextran matrix. The final immobilization level for the active flow cell reached approximately 280 response units (RU). Monoclonal antibody samples were injected at 15 nM for the 60 s with a flow rate of 10 $\mu L \cdot min^{-1}$.

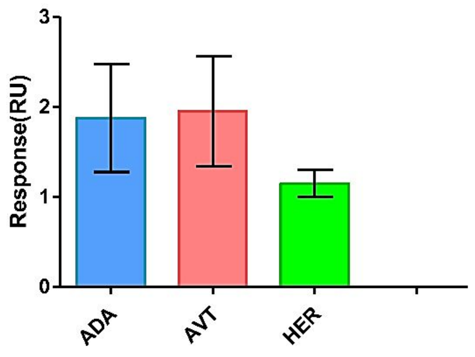

Figure S2 Antibody binding responses were evaluated with EDC/Sulpho-NHS conjugation chemistry for FcγRIa

Table S1. Parameters related to FcγRIa interactions with AVT in different FcγRIa ligand concentrations. The kinetic parameters were calculated by Biacore Evaluation Software using a 1:1 Langmuir binding model.

FcγRIa			
**Concentration (nM)**	**Capture level (RU)**	**AVT Binding response (RU)**	**Chi^2**
28	409±10.7	107.7 ±3.3	0.44±0.05
35	536±24.9	162.8 ±10.0	0.98±0.15
42	658±14.5	224 ±6.01	1.7±0.3
49	718±3.3	262 ±4.9	2.5±0.05

Fc gamma receptor guideline(Cytiva[2]) was applied for the His capture FcγRIa binding assay. The association, dissociation, and flow rate were followed depending on this guideline. FcγRIa ligand capture level was assessed with varying concentrations (28 nM, 35 nM, 42 nM, and 49 nM). Increased concentration of FcγRIa led to increased Chi^2 value in kinetic analysis (Table S1). Also, the literature states that kinetic analysis should be performed with low response levels. Therefore, FcγRIa capture levels were selected as 200 RU with 14 nM FcγRIa concentration.

EDC/NHS and streptavidin-biotin methods were not optimized.

(1) Cohen-Solal, J. F. G.; Cassard, L.; Fournier, E. M.; Loncar, S. M.; Fridman, W. H.; Sautès-Fridman, C. Metastatic Melanomas Express Inhibitory Low Affinity Fc Gamma Receptor and Escape Humoral Immunity. *Dermatol. Res. Pract.* **2010**, *2010* (1), 1–11. https://doi.org/10.1155/2010/657406.

(2) Cytiva. Fc receptor binding assays using surface plasmon resonance https://cdn.cytivalifesciences.com/api/public/content/digi-34141-pdf (accessed May 25, 2022).
